# Cascading and Parallelising Curvilinear Inertial Focusing Systems for High Volume, Wide Size Distribution, Separation and Concentration of Particles

**DOI:** 10.1038/srep36386

**Published:** 2016-11-03

**Authors:** B. Miller, M. Jimenez, H. Bridle

**Affiliations:** 1 Institute for Infrastructure and Environment, School of Engineering, The University of Edinburgh, The King’s Buildings, Edinburgh, EH9 3FG, United Kingdom; 2 Institute of Biological Chemistry, Biophysics and Bioengineering, Heriot-watt University, Riccarton, Edinburgh, EH14 4AS, United Kingdom

## Abstract

Inertial focusing is a microfluidic based separation and concentration technology that has expanded rapidly in the last few years. Throughput is high compared to other microfluidic approaches although sample volumes have typically remained in the millilitre range. Here we present a strategy for achieving rapid high volume processing with stacked and cascaded inertial focusing systems, allowing for separation and concentration of particles with a large size range, demonstrated here from 30 μm–300 μm. The system is based on curved channels, in a novel toroidal configuration and a stack of 20 devices has been shown to operate at 1 L/min. Recirculation allows for efficient removal of large particles whereas a cascading strategy enables sequential removal of particles down to a final stage where the target particle size can be concentrated. The demonstration of curved stacked channels operating in a cascaded manner allows for high throughput applications, potentially replacing filtration in applications such as environmental monitoring, industrial cleaning processes, biomedical and bioprocessing and many more.

Particle separation and concentration has applications in a wide range of different fields: from industrial *e.g.* wastewater treatment^1^,^2^, water purification^3^, microelectronics^4^, chemical processing^5^, fermentation^6^ and filtration; to biomedical and bioprocessing, *e.g.* disease diagnosis^7^,^8^,^9^,^10^. Traditional approaches such as filtration and centrifugation suffer from several drawbacks including being labour intensive and limited by the heterogeneity of the sample^8^. Additionally, centrifugation is bulky, expensive and difficult to automate whereas filtration is prone to clogging^11^. Microfluidics offers a promising alternative for particle separation and concentration. Among many options one passive hydrodynamic approach, known as inertial focusing^12^, provides an excellent means with which to separate and concentrate particles into size fractions combined with high sample throughput^13^,^14^,^15^. Straight channel^16^, serpentine^7^,^17^,^18^ and spiral^19^,^20^ channels have all been explored as well as geometries incorporating trapezoidal or slanted channels^21^,^22^, multiple inlets^19^ for switching particles into a clean fluid stream or side expansions generating microvortices^8^. Inertial focusing (IF) is a rapidly expanding area of microfluidics finding many applications in sample processing of circulating tumour cells^23^, isolation of components from blood^7^,^24^, separation of deformable emulsions^7^, cell cycle enrichment^25^ and flow cytometry^26^ among many others^14^. The use of open channels with no obstacles coupled with relatively high liquid shear helps to reduce the tendency of devices to clog. Another reason for the popularity of IF devices is the high throughput compared to other microfluidics approaches^8^. Though volumes have typically remain on the mL level, recent efforts have been made to parallelise these to increase throughput^27^.

Several applications exist where the sample processing size fractionation of IF would be highly desirable but where process volumes are several orders of magnitude larger than the typical mL. For example in environmental monitoring for pathogens such as *Cryptosporidium*, 1000 L is filtered^28^,^29^,^30^. Other examples include algal dewatering for biofuel production^31^,^32^, to clean circulating oil in vehicles and heavy rotating machinery^33^ and bioprocessing/biotechnology^34^.

As implied above parallelization is oft quoted as the solution to increase throughput, particularly in microfluidics. However, as highlighted in Table S1, there are much fewer examples of successful parallelization strategies in the literature, particularly for large particles separation using inertial focusing (>60 μm in diameter). Removing pathogenic bacteria from blood samples appears to be the application which has received most attention for parallelization in inertial focusing. Mach *et al.*^35^ reported a parallelized approach based on a “Ferris wheel” arrangement of straight channels with 40 devices fed from one inlet. This system processed 30 mL of blood at a flow rate of 8 mL/min and it was proposed that stacking of the single layer Ferris arrangement could enable further throughput increases. Hansson *et al.* also parallelized straight channels for a similar application achieving a higher filtration efficiency of 95% with 4 and 16 channel devices that could be operated at 4 mL/min^16^. Straight and serpentine channels have been considered easier to parallelize^8^,^10^,^18^. Spiral channels offer the advantage of particle focusing at one lateral position usually close to the inner wall until a threshold concentration is reached, though the central inlet location makes parallelization challenging. Some designs have proposed a double inlet to the spiral^24^,^36^ with flow entering on the outer edge of the spiral, with the aim of enabling easier parallelization, although this merely transfers the problem to the outlet.

As Mach noted stacking could enable higher throughput since in-plane parallelization results in an expanded device footprint which can quickly become impractical and this strategy for inertial focusing was demonstrated in a prototype system developed by Parc (personal communication) and in recent work by Warkiani^21^,^27^. However, inlet pressure variance occurs when stacking and attempting to pump from one or both ends of an access port/pipe as the distance from the pressure source is not equal through the stack. Given that flow rate is a critical parameter in determining separation efficiency^12^, we have developed a new stacking approach using a novel toroidal channel design to enable easy access to the inlet and outlets along with a novel manifold to deliver equalized inlet pressure to the stack. The new designs of the IF channel as well as the novel stacking approach, here incorporating 20 devices, has enabled processing of 1 L/min doubling the current highest throughput rate for IF reported recently by Warkiani at 500 mL/min.

Recirculation or multiple passes within IF devices has been proposed previously for increasing purity and yield, having been described by the authors as operating devices in series^7^. However, the same sizes of devices were adopted. Cascading as a strategy has been extended in this work to employ a series of different sized devices to allow for an efficient separation of particles from an initial sample with a particle distribution size ranging over several orders of magnitude (2–300 μm). In addition to cascading, recirculation is necessary to ensure high recovery rates. We demonstrate here the principle of recirculating and cascading to successfully separate and concentrate for the first time small particles (down to ~4 μm) from a mixture with a large range of particle sizes (up to 300 μm), providing an alternative to filtration for complex samples. The devices proposed in this work are produced using laser-cutting technologies, which allow rapid design changes and low cost manufacture in comparison to standard photolithography. Finally we propose that a combination of the stacking, recirculation and cascade strategy will enable large volume processing of samples with a large particle size range, opening up inertial focusing systems to a wider range of industrial and biotechnology applications.

## Results

**Figure 1:**
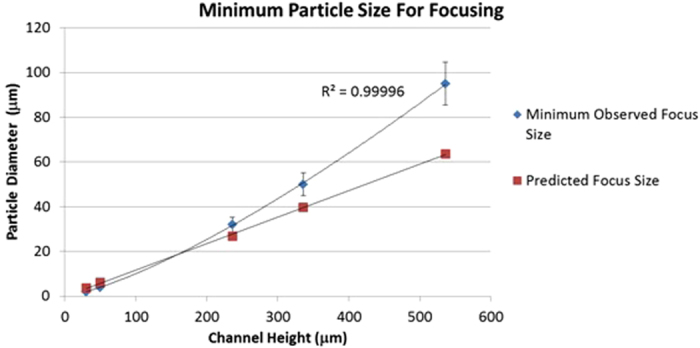
Observed minimum focusing sizes in relation to the spiral channel height and particle size (blue diamonds) compared against predictions (red squares) established by the hydraulic diameter relationship (a/D_h_ >  0.07^15^). Error bars of +/− 10% result from the bead size distributions used for these experiments (*cf.* beads characteristics in Table S4) with the data shown being based on the mean particle size reported by the manufacturer. The location of single particles is imaged using a high speed camera (cf. Fig. S5) or by visual observation for the larger particles (>75 μm) as presented in the video in SI. Particles are “observed to focus” when they align close to the inner wall of the spiral with 100% of particles going into the focused outlet based on 100 recorded images (number of particles detected for each case>200).

A series of curved devices with different designs (described in Fig. S1 and Table S3) were investigated for creating cascaded and stacked systems with performance characterized *via* imaging of particles within the device outlet channels and analyzing samples collected from the outlets using a laser diffraction particle size analyzer (Mastersizer 2000, Malvern Instruments). All the designs presented a rectangular cross-section and utilized two outlets; the one closest to the inner wall being denoted the focused outlet since the focused particles, above a certain critical diameter are collected here. The critical diameter is function of the channel geometry and has been empirically estimated by *a*/*D*_*h*_ > 0.07^15^, where *a* is the particle diameter and *D*_*h*_ is the hydraulic diameter of the channel (Fig. 1).

### Performance characterisation with spiral channels

**Figure 2:**
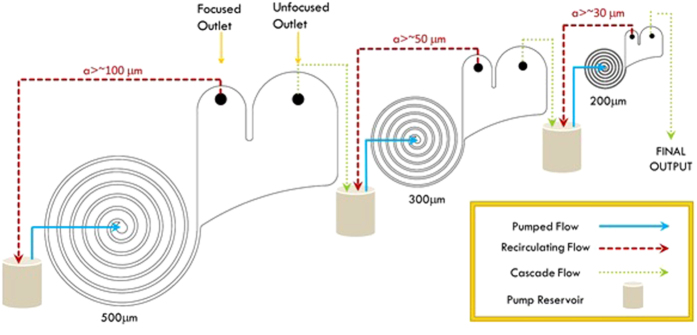
Cascade flow diagram. 3 spiral rectangular channels (500, 300 and 200 μm high) are cascaded to fractionate a mix of particles with a wide range size as described in Table S5. Each spiral presents a similar design, scaled down to focus different particle sizes (~100 μm for the 500 μm high spiral, ~50 μm for the 300 μm high spiral and ~30 μm for the 200 μm high spiral). The initial sample volume (500 mL) is injected through the 500 μm high spiral (blue arrow) and split equally into two outlets. The focused outlet contains focused particles while smaller particles are homogeneously distributed in the two outlets. The focused outlet is continuously re-injected at the inlet of the spiral until the desired volume is reached (~50 mL in this case). A 50 mL sample is collected from the unfocused outlet for measurements, the remainder of which is then injected through the 300 μm high spiral and recirculated in a similar manner until the volume at the focused outlet reaches ~50 mL. The same process is repeated with the 200 μm high spiral (final volume at the focused outlet ~50 mL). The flow rates were 22, 12 and 7 mL/min respectively.

A set of spiral designs with 6 turns and 2 outlets (as illustrated schematically in Fig. 2) were produced using a laser cutter with the dimensions given in Table S3 (first three rows of the table; 500, 300 and 200 μm high spiral channels with 1:6 aspect ratio). All spiral designs have the same structure (number of loops, design and number of outlets and aspect ratio) but were scaled to target the focus of a different particle size. The standard spiral designs were tested first to establish a benchmark for performance. A further two scaled device sizes were tested to determine the minimum focusing sizes of particles in 50 μm and 30 μm high spiral designs. These devices could be applied at the end of a cascading strategy involving the first three spirals to shift from “filtration”, and removal of larger particles, to concentrate small particles of interest (~2–6 μm) from a mixture containing particle sizes between 2–300 μm. It is shown in Fig. 1 that the minimum size for focusing particles of diameter *a* observed in these scaled designs did not follow the rule commonly used to predict the sizes at which focusing generally occurs (*a*/*D*_*h*_ > 0.07^15^) with discrepancies of up to 50%. A new relationship between minimum focusing size and channel height has been observed with these scaled devices, which is described by the following equation


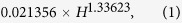


where *H* is the channel height (in μm).

This relationship, though purely empirical, may be useful for precisely targeting specific sizes of particles, provided that the channel design conforms to the ones produced for this work. Precise details for reproducing the spiral designs can be found in the SI.

### Cascading and recirculation set-up

The use of a cascade of sequentially decremented scaled geometries reduces the consequent size of particles which can be focused at each stage. By choosing overlapping focusing size ranges, the efficient removal of particles large enough to clog the smaller geometries is ensured. The net effect is to fractionate and concentrate particle size ranges from a broad spectrum particle size distribution starting with the largest and working down to the smallest particle sizes. Each stage of the cascading strategy (Fig. 2) has been tested individually, with single bead sizes as well as mixed bead sample inputs.

**Figure 3:**
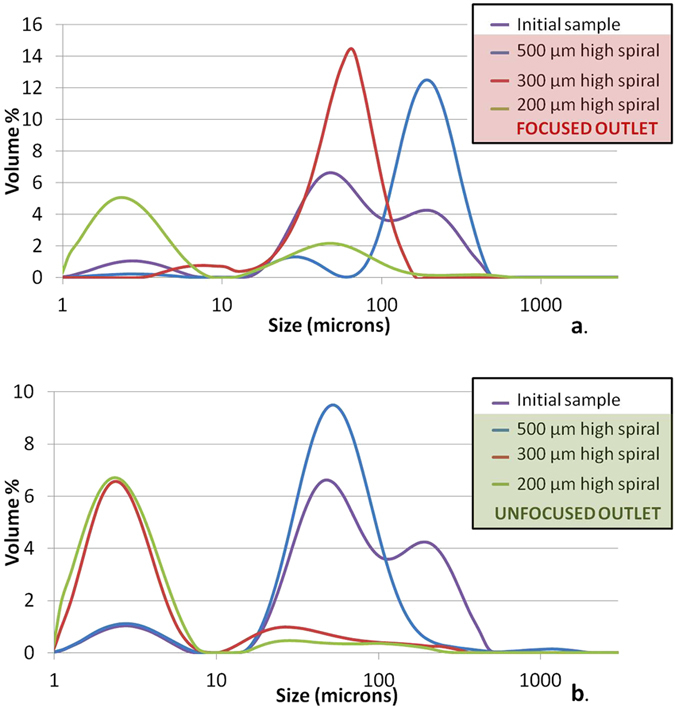
Particle size distribution results for a three stage cascade (500, 300 and 200 μm high spirals) operated sequentially after approximately 2 recirculations per device (the focused outlet is continuously re-injected in the spiral until it runs down to 50 mL left in the inlet reservoir). The bead sizes ranged from 1 μm to 300 μm with concentrations as indicated in Table S5. Initial sample particle size distribution and subsequent distributions present at the focused (**a**) and unfocused (**b**) outlet from each device profile size. Volume % corresponds to the volume of particles detected for a particular size range divided by the total volume of particles detected (*cf*. Performance Characterization section for further details).

Figure 3 illustrates the results of testing a three stage sequenced cascade (top 3 devices in Table S3) with a mixed bead sample between 1 μm and 250 μm (Table S5) from a 500 mL sample with ~2 continuous recirculations per device. Further details on the operation of the cascade and on the notion of recirculation are proposed in Fig. S2. Successful particle focusing is shown in Fig. 3-a, where the volume percentage of larger (>100 μm) particles is significantly increased in the focused outlet of the 500 μm high spiral compared to the initial sample. Figure 3-b then confirms the removal of these particles in the unfocused outlet of the first cascade stage (500 μm high spiral) and similar data is observed for each of the next two stages. Fragmentation of some of the beads introduced to the cascade were observed visually and evidence of this was captured using the high speed camera (Fig. S3). The fragmentation is clear in Fig. 3 where the particle size distribution at the unfocused outlet of the 200 μm high spiral shows a broad distribution and low detection count of larger particles. The source of this fragmentation is most likely high shear forces within the pump and it is recommended to use a progressing cavity pump to mitigate this issue. It can be noted that for smaller inertial focusing channels with similar profile heights (30 and 50 μm high), 100% recoveries have been reported in previous works where syringe pumps have been used^29^,^30^,^37^. However, such pumps cannot be used for recirculating continuously the sample.

In order to quantify the focusing performance a plot of the area under the curves shown in Fig. 3 for certain particle size classes were computed (Fig. S4). The data indicates that approximately 95% of the particles above 95 μm (*i.e.* those which focus in this device) are found in the expected focused outlet after ~2 recirculations in the 500 μm high spiral indicating a high degree of concentration/removal of the large particles. The 300 μm high spiral performed equally well, enriching the larger particles (>50 μm) 19-fold, and achieving 96% collection into the focused outlet. As detailed in Fig. S2, performances could be further improved by increasing the number of recirculations. In the 200 μm high spiral device some large beads (>250 μm) were recorded by the laser diffraction particle size analyzer as seen in Fig. S4, although this anomaly, given the fact that these beads are larger than the channel height and would thus clog the device, might well be explained by the bead fragmentation described above, and result from fragmented beads. Cascading in this way thus offers a means of sequential removal of larger particles as an alternative to traditional filtration concentrating the particles above the critical focusing diameter into the focused outlet and enabling high recovery of the smaller particles below this diameter into the unfocused outlet.

### Concentration, recovery and time required

The concentration factor, *C*, achieved by a single device layer is given by Equation 2


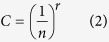


where *n* is the number of outlets and *r* is the number of recirculations as defined in Fig. S2. Therefore, a two outlet design like those presented here would reduce 100 L to 1.56 L in the focused outlet with 6 recirculations (*C* = 0.0156) although this will be limited by the dead volume of the system. Since small particles remain unfocused the percentage recovered into the unfocused outlet after a certain number of recirculations is 1-*C* such that we could expect a recovery rate of 0.98 or 98% from 6 recirculations. Higher recoveries can be reached by increasing the number of recirculations *r* although issues of dead volume, and any particle loss within the system, act to reduce this number. Previous work by Warkiani *et al.*^24^ has reported recoveries of similar magnitude (85%).

The time *t* required for sample processing can be calculated using Equation 3


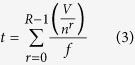


where *V* is the sample volume, *f* is the flow rate and *R* is total number of recirculations applied. Therefore using the stacked system which runs at 1 L/min, detailed in the next section, a sample of 100 L could be reduced to 1.5625 L after 6 recirculations in approximately 3.25 hours (193.75 mins).

**Figure 4:**
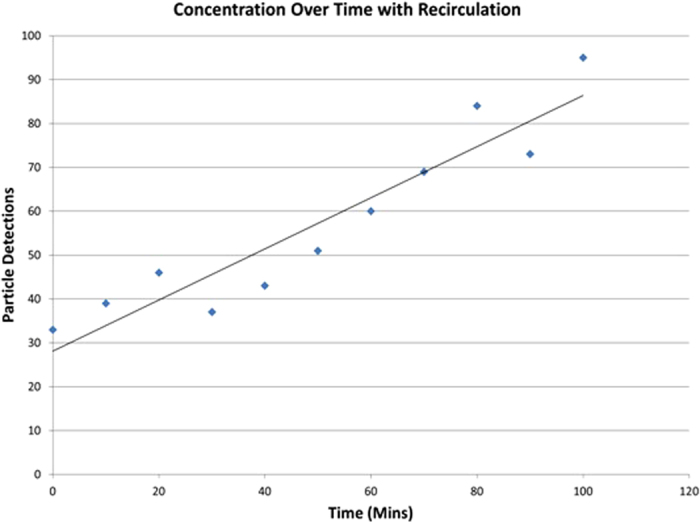
Particle detection over time as counted by high speed camera imaging of particles in the focused outlet (*cf.* Fig. S5). The data points shown are an average particle count from 100 images recorded every 10 minutes using orange beads (75–90 μm) recirculating in the 200 μm high spiral device at a flow rate of 6.5 mL/min.

The concentration effect was studied using 75–90 μm orange beads in the 200 μm high spiral design. Results, based on image processing of particle trajectories at the outlet as detailed in Fig. S5, are shown in Fig. 4. A 450 mL inlet sample was recirculated over 100 mins down to 125 mL. The expected linear evolution of concentration over a series of 1.848 recirculations is clearly indicated in the figure, though the detected overall concentration factor falls slightly short of what would be expected, with a value of 2.9 (corresponding to 1.536 cycles) versus 3.6 expected. Fragmented beads (Fig. S3), depending on their orientation, were not recognised by the algorithm which is thought to explain this discrepancy. The drop observed after 30 minutes is likely to be due to an accumulation of beads in the outlet tubing before being expelled back into the reservoir. This temporal variation is likely to be attributable to non uniform return to the reservoir impacting the number of particles being detected.

### Stacked system

Two designs, the semi-circular and toroidal designs (last two rows in Table S3), were developed in order to enable the insertion of a manifold for stacking. These were designed to have the same length, and therefore ideally the same focusing behavior, as the spiral of corresponding channel height.

**Figure 5:**
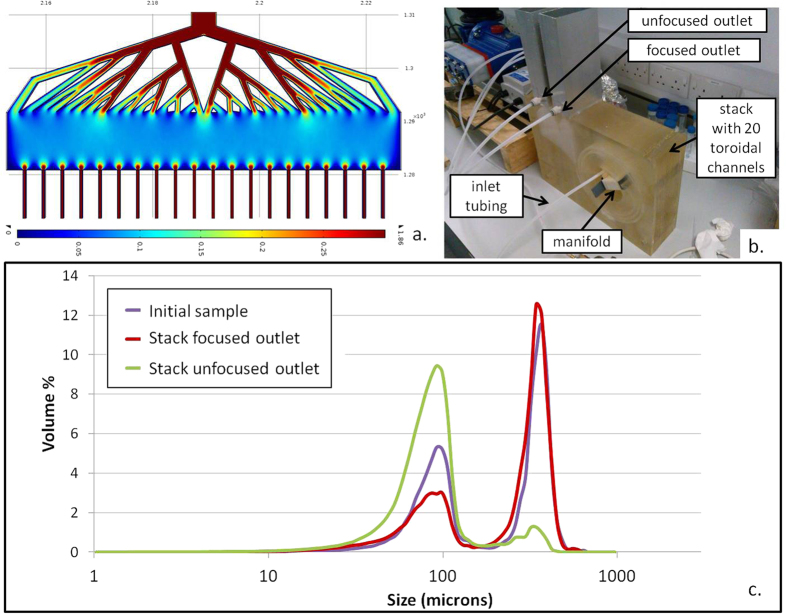
(**a**) Inlet manifold simulation velocity field (in m/s) at mid-plane. The width of the manifold is 9.3 cm. (**b**) A photo of the stacked system. The stack consists of 20 rectangular toroidal channels (500 μm high, 1:6 aspect ratio). The manifold described in Fig. 5-a is inserted at the centre of the stack (white block) to ensure an homogenous fluid distribution between the different layers. Two aluminium box sections are sealed at the top of the system to collect the liquid coming out from the focused and unfocused outlets respectively, with gravity equalising the back pressure at the outlets. Two populations of particles (as described in Table S6) are diluted in 7.05 L of tap water and processed through the stack at 1 L/min over 1.7 recirculations. (**c**) Corresponding particle size distributions at the inlet (purple curve), focused (red curve) and unfocused (green curve) outlets.

The challenge with stacking spiral channels is that fluid delivery is from either the top or the bottom in a vertical array of devices, and therefore flow rates will vary between layers (see the “Heterogeneities in fluid distribution in stacked spiral micro-channels” section in the SI). Inertial focusing depends upon flow rate and if it is too low a focusing effect is not observed. Therefore, variation in flowrate could negatively impact upon the performance of a stacked set of channels. To overcome these issues a manifold approach to device stacking has been developed. The modular manifold was designed, inspired by a technique for ensuring highly uniform flow across an open linear section^38^, and in our case adapted to evenly distribute pressure and flow rate across all channels of the stack. To check the adapted design’s expected performance before production, COMSOL modelling was undertaken to predict the flow rate distribution across the 20 manifold outlets/20 device layer inputs (Fig. 5-a). Table S3 shows that a semi-circular approach allowing easy access of the manifold to the inlets (Fig. S1-Semi-circular design) was unsuccessful at focusing in the range of flow rates trialled. It is thought that the increased radius of curvature combined with the upper limit on pumping capacity hindered the formation of the secondary Dean Flows, thereby resulting in no focusing. Therefore, the toroidal design was selected and a stack of 20 of the 500 μm high toroidal inertial focusing channels was produced. The stacked system is shown in Fig. 5-b with further details provided in the SI (Fig. S7).

The 20 layer stacked toroidal spirals device was tested with ~250 μm (blue) and ~45 μm (red) beads at a flow rate of 1 L/min. The focusing of particles into the outlet closest to the inner wall was clearly visible but to further quantify the performance of the system particle size distributions were measured using the laser diffraction particle size analyzer to compare the inlet sample with those collected from the outlets. A total of 7.05 L of sample was circulated until 2.51 L was remaining in the inlet reservoir (~1.7 recirculations). Figure 5-c shows the resultant particle distributions at both the focused and unfocused outlets with the initial sample superimposed. The results indicate the level of performance of separation commensurate with expectations (very low concentration in large particles >100 μm in the unfocused outlet; particle size distributions being expressed in volumes, there is an emphasis on large particles), with some fragmentation still evident. According to individual particle counting measurements, ~88% of large particles were concentrated in the focused outlet while the unfocused outlet contained ~73% of the smaller particles after 1.7 recirculations.

## Discussion

Here we show how a cascading and recirculating strategy can successfully separate and concentrate small particles from a mixture with a large range of particle sizes (2–300 μm) with enrichment factors of above 19, that could be improved by further recirculation steps. By sequentially removing the largest particles into a highly concentrated sample, the particle range is narrowed until the target particle size at which point this size is focussed and concentrated. This technique offers an alternative to filtration with the additional advantage that unlike traditional filtration processes which concentrate all particles above a given cut-off a relatively narrow size band of particles can be extracted from a complex mixture.

We demonstrate larger scale inertial focusing than have been previously shown in the literature and observe at larger (>300 μm) channel heights that the critical size at which a particle becomes focussed is higher than predicted from the established empirical formula. A similar deviation is also observed at smaller channel heights, although in this case smaller particles than expected are focused. We propose a new relation between critical focusing particle diameter and channel height for spiral systems similar to those produced in this paper. This will assist in the design of such systems for other applications.

Furthermore, we show the potential of stacking to increase the throughput of inertial focusing systems to industrially relevant sample volumes. In particular we have demonstrated a stack of 20 devices operating at 1 L/min, setting a new precedent for microfluidics throughput. This was achieved through the use of a novel toroidal inertial focusing design. The stacking approach presented enables even distribution of pressure and flow rates throughout the layers ensuring maintenance of performance and potentially enabling a larger number of devices to be integrated into a system. We believe this approach could be useful for environmental monitoring as well as industrial filtration applications and bioprocessing markets.

## Materials and Methods

### Device Designs and Manufacture

The single spiral devices used in the cascade (Fig. 3) were all manufactured in PMMA using an Epilog Mini 24 CO_2_ laser cutter, tuned individually for each material thickness, to pattern device layers. PSA1589F adhesive transfer tape (18 μm thickness) was applied to both sides of the device layer material prior to patterning. Assembly was achieved by removing the tape backing and sandwiching between a ported layer and a substrate layer. Connections for the cascaded devices were achieved with generic 1/8″ bspt barbed tubing connectors and 2.4 mm ID tygon tubing. To run the spiral channels a 12-roller Watson-Marlow Peristaltic pump was used. The semi-circular device was also produced using this technique. A height of 200 μm was the maximum which could be manufactured due to constraints of the laser cutter workbed relative to the length of channel required to be directly comparable to a spiral of the same height.

For the devices with channel height of 30 μm manufacture was outsourced to the commercial company Epigem Ltd. From designs produced using AutoCAD Epigem manufactured the systems in Epoxy and PMMA. These devices were run using a WPI AL1000-220 syringe pump and 5 mL BD Lure Lock syringes. 50 μm devices were laser micro-machined and bonded using a hybrid solvent/plasticiser low temperature press bonding method.

The stack of toroidal 500 μm high spirals (Fig. 5-b) was produced by the individual manufacture of each device layer with assembly taking place through a jigged alignment process and finally bonding was achieved using high tack adhesive transfer tape. For the stack 1/8″ bspt to 6 mm push fit connectors were used in conjunction with ¼″ LDPE tubing. Device and substrate layers were patterned using a CNC Laser Cutter exploiting the larger workbed. This method was also used to produce the single layer 500 μm high toroidal channel used to confirm the focusing performance of this design.

Further details regarding each designs tested in this work are available in the SI (Tables S2 and S3, Figs S1 and S7).

### Manifold manufacture

The manifold, presented in Fig. 5-a, was manufactured in nylon using a 3D printer from a model created in AutoCAD 2012.

### Comsol simulation

A velocity field of the mid z-plane section was generated using the laminar flow solver on the geometry of the manifold imported from the AutoCAD 3D model at 1 L/min flow rate with 20 inlet sections of the same geometry as the 500 μm high device inlets.

### Chemicals and Beads

All of the beads above 5 μm were sourced from Cospheric LLC, with the exception of the turquoise and red beads which were obtained from Phosphorex. The beads of size 5 μm and below were sourced from Magsphere. All bead details are given in Table S4. Tween 20 (Sigma-Aldrich) was added to the sample to reduce aggregation of beads due to static build up on the polystyrene beads. A magnetic stirrer was used in the inlet reservoir to agitate the sample to prevent settling of the beads (Fig. S2).

### Performance Characterization

Particle size measurements were obtained in triplicate using a laser diffraction particle size analyzer (Mastersizer 2000, Malvern Instruments) to analyse the inlet samples and the subsequent samples collected from the outlets. Particle size distributions measured by the Mastersizer are expressed in volume percentage.

A high speed camera setup (CCD ProgRes, Jenoptik, GmbH), mounted to an inverted microscope (Nikon, x10 or x25 magnification), was used to observe the evolution of concentration over time in a single layer system with a single bead size population. In the focussed outlet a batch of 100 photos was collected at 10 min intervals. Data was analysed in MATLAB and beads were detected based on intensity differences between background and particles using thresholds (*cf.* Fig. S5).

## Additional Information

**How to cite this article**: Miller, B. *et al.* Cascading and Parallelising Curvilinear Inertial Focusing Systems for High Volume, Wide Size Distribution, Separation and Concentration of Particles. *Sci. Rep.* **6**, 36386; doi: 10.1038/srep36386 (2016).

**Publisher’s note**: Springer Nature remains neutral with regard to jurisdictional claims in published maps and institutional affiliations.

## Supplementary Material

Supplementary Movie

Supplementary Information
