# Supplementary material for: Cascading and Parallelising Curvilinear Inertial Focusing Systems for High Volume, Wide Size Distribution, Separation and Concentration of Particles
Source: Sci Rep. 2016 Nov 3;6:36386. doi: 10.1038/srep36386 (PMC5093461; doi:10.1038/srep36386)
Supplement: Supplementary Information [file srep36386-s2.pdf]

Supplementary Information

Cascading and Parallelising Curvilinear Inertial Focusing Systems for High Volume, Wide Size Distribution, Separation and Concentration of Particles

Miller, B.[1], Jimenez, M.[2] and Bridle, H.*[2]

[1]Institute for Infrastructure and Environment, School of Engineering, The University of Edinburgh, The King's Buildings, Edinburgh, EH9 3FG, Scotland

[2]Institute of Biological Chemistry, Biophysics and Bioengineering, Heriot-watt University, Riccarton, Edinburgh, EH14 4AS, Scotland

Miller,B. and Jimenez,M. are joint primary authors

*corresponding author: h.l.bridle@hw.ac.uk

State of the art

Authors	Short description	Flowrate	Particles
Mach and Di Carlo (2010)[1]	40 straight micro-channels placed as a radial array (60x20 μm²)	8 mL/min	Blood cells (RBCs ~6μm, platelets ~3μm, WBCs ~10μm) Beads 7.9 μm
Khoo *et al.* (2014)[2]	3 stacked spirals (155x500μm²)	1.5 mL/min	Circulating tumor cells (~15μm) Beads 6 and 15μm
Lee *et al.* (2015)[3]	10 loops of helical microchannel (250-500x1000 μm²)	5 mL/min	*E.coli* (~1μm)
Warkiani *et al.* (2016)[4]	3 stacked spirals (170x500 μm²)	300 μL/min (cell sample)	Circulating tumor cells Blood cells Beads 6, 10, 15 μm
Hur *et al.* (2009)[5]	256 parallel straight channels (37x16 μm²)	2,5 mL/min	Blood cells Beads 7.9 and 9.9μm
Zhang *et al.* (2014)[6]	8 parallel serpentine channels (42x200 μm²)	2,8 mL/min	Blood cells Beads 3 and 10μm
Hansson *et al.* (2012)[7]	4 and 16 parallel straight channels (30x80 μm²)	0,8 mL/min (4 devices) 3,2 mL/min (16 devices)	Beads 10μm
Rafeie *et al.* (2016)[8]	16 channels (8 spirals in each layer) (70-50x500 μm²)	24 mL/min	Blood cells Beads 3, 4.2 and 9.9μm
Martel et al. (2015)[9]	10 parallel 10x devices that feed into a single 50x devices (50-200x52 μm²)	4 mL/min	Beads 5, 10 and 15 μm

Jimenez and Bridle (2016) [10]	6 loops spiral microchannel (30x170 μm²)	1.5 mL/min	Cryptosporidium parvum (4-5μm) Giardia lamblia (10-15μm)
Warkiani *et al.* (2015)[11]	84 parallel spiral channels (600x80-130 μm²) (450x30-70 μm²)	500 mL/min	CHO cells (10-20μm) Yeast cells (3-5μm) Beads 10 and 15μm
Guan *et al.* (2013)[12]	8 loops spiral trapezoidal channel (600x80-130 μm²)	Up to 6 mL/min	Beads up to 26,9μm
Hasni *et al.* (2011)[13]	5 loops spiral microchannel (220x500 μm²)	3 mL/min	Beads 40 and 60μm

Table S1. Examples of publications considering inertial focusing for separating particles at high throughput.

Single Device Design and characteristics

Using the helix command to draw two spirals to form the functional channel in AutoCAD with the following parameters

- All spirals are scaled down from a single design of the 500 μm high device of 6:1 aspect ratio
- Number of turns = 6
- Inner Diameter = 0, 3 (mm)
- Outer Diameter = 66, 69 (mm)
- Z height = 0 (mm)*
- EXPLODE command to convert spiral object to a spline (precision 50, one at a time, cannot operate on multiple spirals)
- SPLINEDIT command to convert to polyline
- Draw and connect ports and outlet design/ports, then BOUNDARY command to create a single closed polyline suitable for laser-cutting/mask manufacture

The SCALE command can then be used to scale the design to the appropriately to the thickness of material/desired height.
OVERKILL and PURGE commands are also used to remove unwanted objects that may be hidden.
A similar procedure was used for the toroidal designs.

**Z height is zero as only a 2D path is required to instruct the laser cutting table to pattern the channels into material of known thickness*

Table S2. Procedure for designing the channels using AutoCAD.

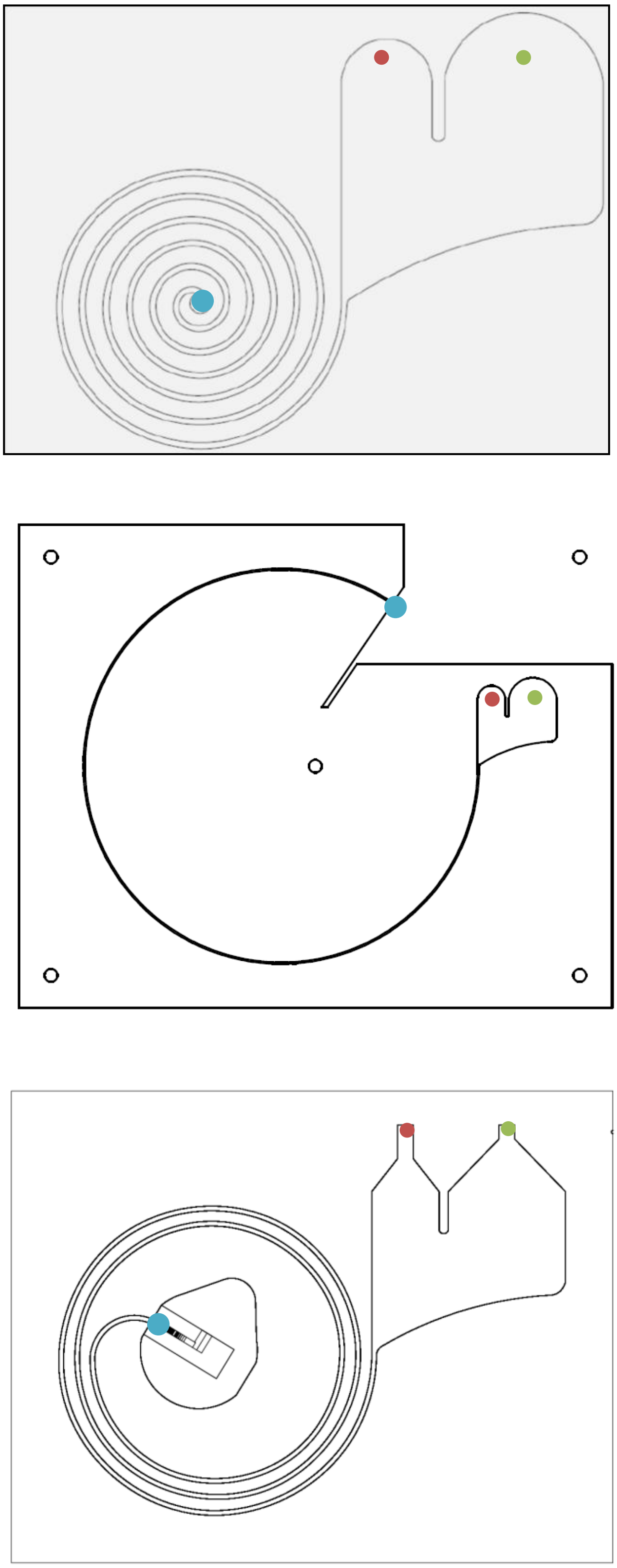

SPIRAL DESIGN
Channel height=536 μm
Aspect ratio=1:6
Shape channel=rectangular
Number of turns=6
Length=124 cm

SEMI-CIRCULAR DESIGN
Channel height=236 μm
Aspect ratio=1:6
Shape channel=rectangular
Number of turns=0.8
Length=50 cm

TOROIDAL DESIGN
Channel height=536 μm
Aspect ratio=1:6
Shape channel=rectangular
Number of turns=2.5
Length=125 cm

Figure S1. Designs of spiral, semi-circular, and toroidal channels tested in this work. Blue dots correspond to the inlet of the channel and red/green dots to the focused and unfocused outlets respectively. Associated dimensions are reported in Table S3.

Device	Width	Height	Channel Length	Expected to Focus (a/D_h>*0.07*[14])	Observed to focus
**500 μm high cascaded spiral**	3 mm	536 μm	~124cm	64 μm	~95 μm
**300 μm high cascaded spiral**	1.8 mm	336 μm	~78 cm	40 μm	~55 μm
**200 μm high cascaded spiral**	1.2 mm	236 μm	~50 cm	27 μm	~32 μm
**50 μm high spiral**	0.3 mm	50 μm	~12.4 cm	6 μm	4 μm
**30 μm high spiral**	0.18 mm	30 μm	~7.8 cm	3.6 μm	2 μm
**Semi-circular channel**	1.2mm	236 μm	~50 cm	27 μm	No Focus (150-180 μm)
**500 μm high toroidal channel**	3 mm	536 μm	~125 cm	64 μm	~95 μm

Table S3. Summary of the different IF devices presented in this paper and their respective observed minimum focusing size. The top set of three devices were used in the cascade (Fig. 2 and 3). The 200 μm high spiral was used to investigate concentration performance over time (Fig. 4). The top 5 spirals were used to investigate the relation between the minimum particle size focused and the channel height (Fig. 1). Note that some observed focusing sizes are approximate due to bead size distributions (*cf.* Table S4). The data reported in the final column is the mean size of the particle as reported by the manufacturer. "Observed to focus" refers to a visualization of a focused stream of beads using a high-speed camera. A video is also available in the SI where focusing of large particles is clearly visible. The bottom two channels were used to find an alternative design to enable more effective stacking (Fig.5) and the toroidal channel was selected as it was observed to behave in a similar manner to the spiral of equivalent length and additionally offered a smaller device footprint.

Beads characteristics

Colour (Manufacturer)	Density (g/cc)	Size Range (μm)
Green (F) (Magsphere)	1.0	1
Green (F) (Magsphere)	1.0	2
Green (F) (Magsphere)	1.0	3
Orange (F) (Micromod)	1.0	4
Green (F) (Cospheric)	1.3	1-5
White (Cospheric)	1.3	10-27
Red (Cospheric)	0.98	38-45
Violet (Cospheric)	1.01	53-63
Turquoise (Phosphorex)	0.99	71 - 79
Orange (Cospheric)	1.0	75-90
Red (Phosphorex)	0.99	225-275
Yellow (Cospheric)	1.0	150-180
Blue (Cospheric)	1.0	250-300

Table S4. Specifications of the beads used for all tests. (F) indicates fluorescent beads.

Cascade experiments-characterisation

Beads (μm)	Mass (g)	% volume
Green, 1-5	0.0731	9.8%
White, 10-27	0.0749	10.3%
Violet, 53-63	0.1343	24.1%
Orange, 75-90	0.1058	19.2%
Yellow, 150-180	0.0797	14.3%
Blue, 250-300	0.1245	22.3%

Table S5. Inlet masses of beads for cascade experiments. The % volume corresponds to the volume of a class of beads divided by the total volume of beads in the sample. The overall volume fraction of beads, in a starting volume of 500 mL, is ~0.1%.

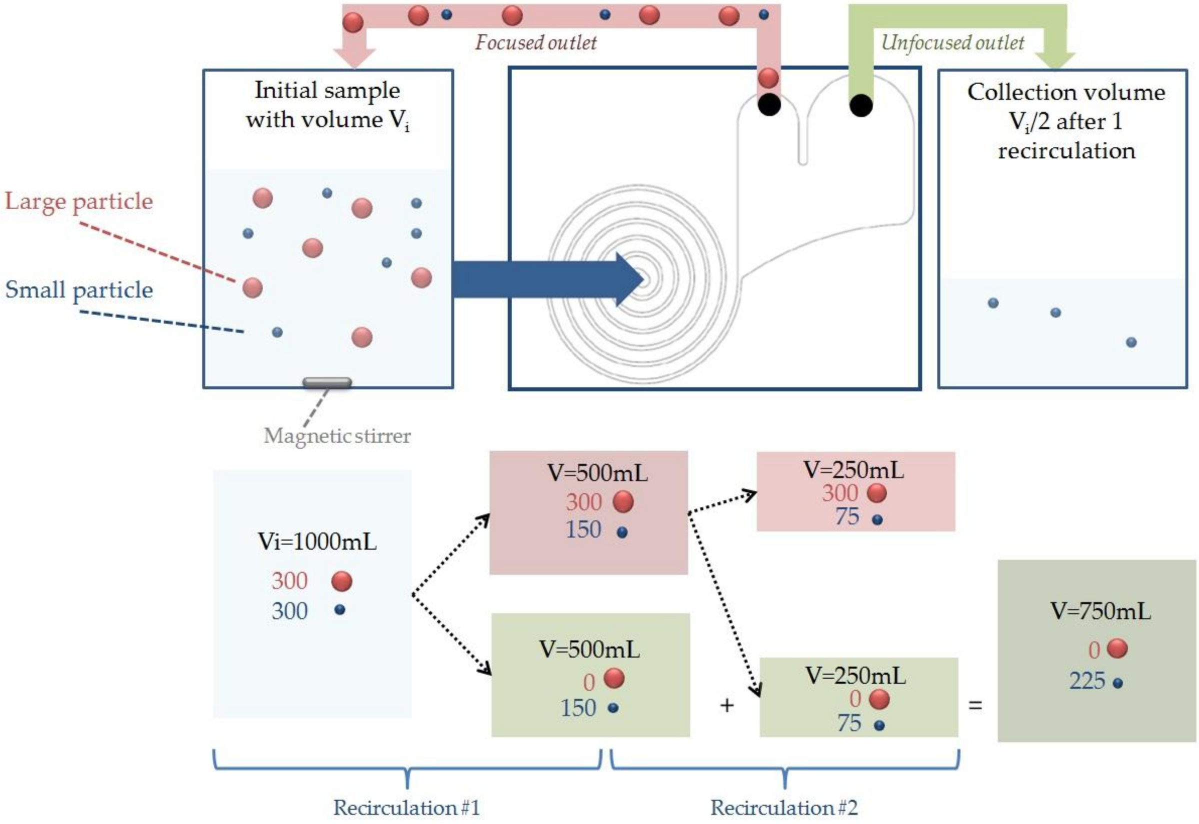

Figure S2. A sample of initial volume V_i is processed through the spiral channel and split into two outlets. The focused outlet containing particles larger than the critical diameter (function of the channel geometry) and half of the smaller particles is continuously re-injected at the inlet of the system for recirculation until the desired concentration is reached. One recirculation is defined as the time required to process the initial volume through the system. In the example presented here, the first recirculation is reached when 1000 mL has been processed (500 mL for recirculation 2, 250 mL for recirculation 3, etc.); the final volume at the unfocused outlet after 2 recirculations being 750 mL with 225/300=75% of the small particles collected. The number of recirculation defines the recovery of small particles; here for instance 4 recirculations would lead to 937.5 mL collected at the unfocused outlet with 94% of the small particles.

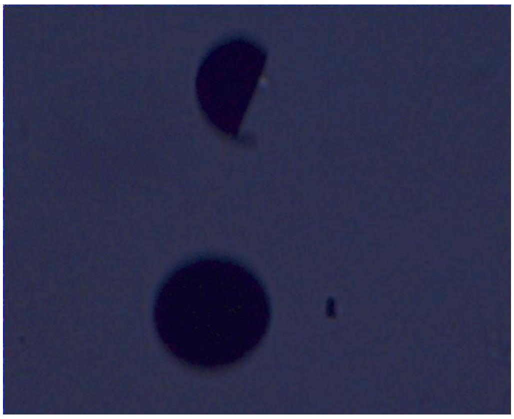

Figure S3. High speed camera image of fragmented beads (Orange beads 75-90 μm in diameter).

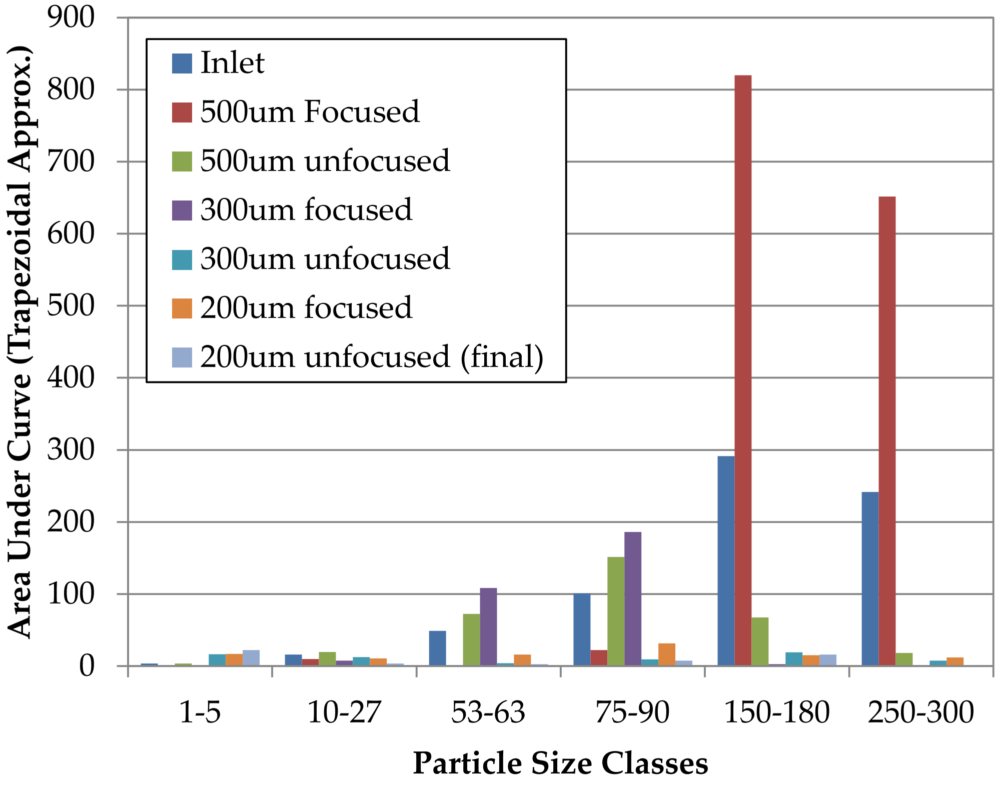

Figure S4. Particle size distributions in each outlet of the cascade experiment are measured using a laser diffraction size analyser (MasterSizer 2000, Malvern Instruments). Raw data are presented in percentage volume as depicted in Fig. 3. Here the corresponding area under the curve is estimated for different particle size classes (*cf*. Table S1) using a trapezoidal approximation.

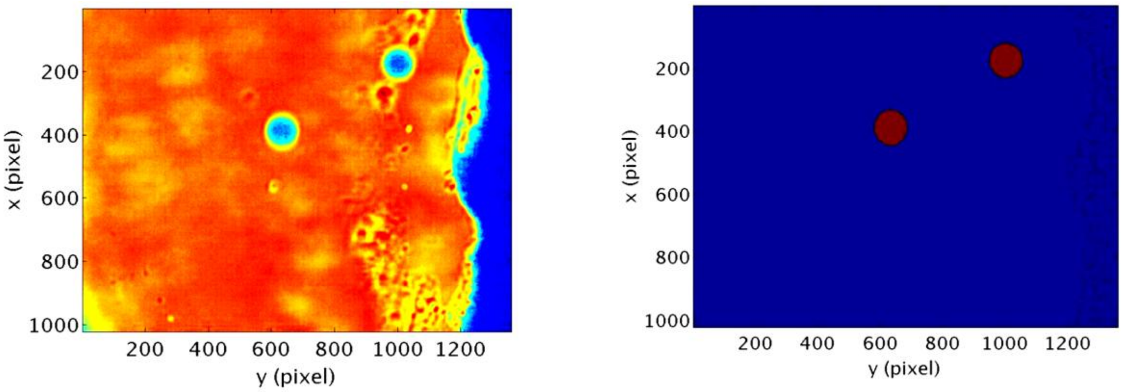

Figure S5. Image processing for particle detection. Raw images are recorded using a high-speed camera and processed in Matlab (left) based on intensity-gradients for detecting the wall (blue zone with y>1200 pixels) and beads (right).

Heterogeneities in fluid distribution in stacked spiral micro-channels

A schematic representation of 10-stacked spirals is shown in Fig. S6-b. Each spiral has a design similar to the one described in a previous work (Jimenez and Bridle, Journal of Microbiological Methods. 126, 8–11, 2016). The sample exits the syringe at a known flowrate, flows through the upper layer of the set-up to reach the middle of the spirals and be distributed vertically from the first spiral at the top to the tenth one at the bottom. In order to detect any discrepancy in the fluid distribution, particle image velocimetry measurements have been performed. The right hand side of Fig. S6 maps the velocity profile of 1 μm particles in the first (top) and last channel (bottom) layer of the stacked configuration. Notions of "first" and "last" channels refers to the order under which the sample is dispersed from the inlet. Although these two velocity profiles look very similar with a noticeable deceleration when the channel widens and a uniform velocity profile in each one of the four outlets, the range of velocities is almost twice as low in the last layer of the stacked configuration than in the layer closest to the sample injection. Assuming a linear correlation between the applied flowrate and the measured velocity profile inside the channel, it can be expected from the experiment detailed in Fig. S6-a (at 500 μL/min) an average velocity of ~2 x 0.21 = 0.42 m/s near the channel outlets for an applied flowrate of 1000 μL/min. The first layer of the stacked configuration presents an average velocity slightly higher than this estimation (~0.49 m/s) while the last layer only reaches ~0.26 m/s. Similar experiments have been carried out in the fifth layer of the stack and presented results similar to the first layer (average velocity in the outlets ~0.45 m/s).

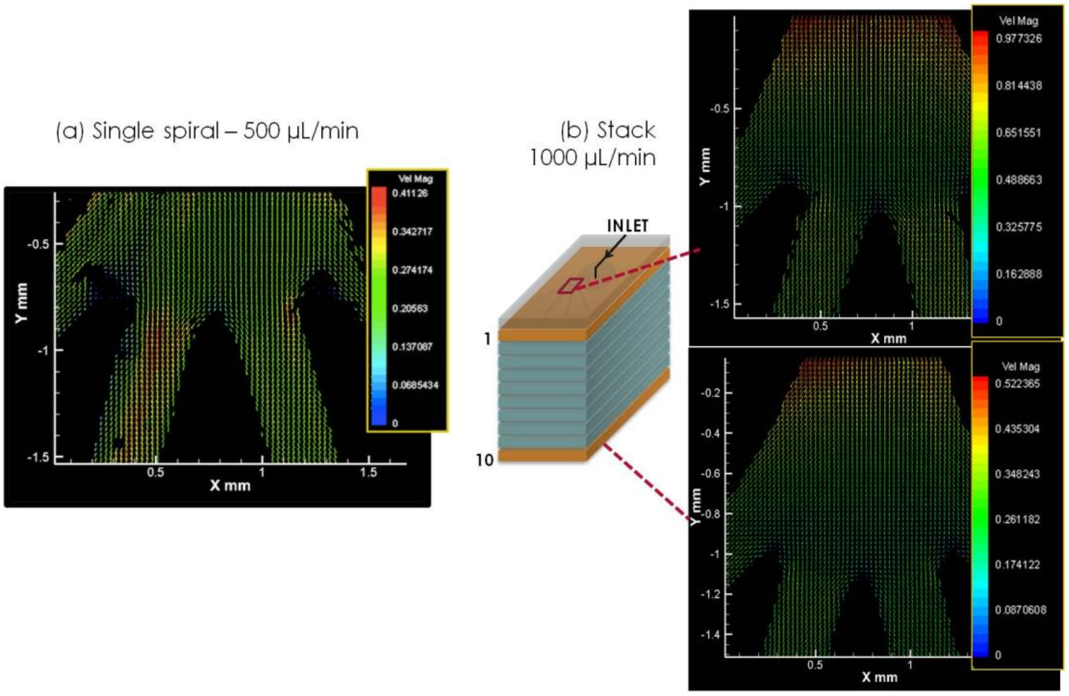

Figure S6. Particle image velocimetry performed near the outlets of **(a)** a single spiral channel using unfocused 1 μm red fluorescent beads and an applied flowrate of 500 μL/min; **(b)** 10 stacked spiral channel using unfocused 1 mm beads and an applied flowrate of 10mL/min (ideally 1000 μL/min per layer). The top (resp. bottom) plot maps the velocity profile in the top (resp. bottom) spiral channel of the stack. Colorbars correspond to estimated velocities expressed in m/s.

A factor 2 in fluid velocities between layers might strongly impact the focussing behaviours of particles and alter the separation efficiency of the system. This was observed with 2 µm beads being collected in two different outlets at 10 mL/min while a 100% separation efficiency in a single outlet was observed in a single layer configuration. This phenomenon might challenge the stacking of an extremely high number of spiral channels unless new designs arise capable of uniformly distributing the sample.

Stacked system

Stack of 20 toroidal channels (Top view)

(Side view)

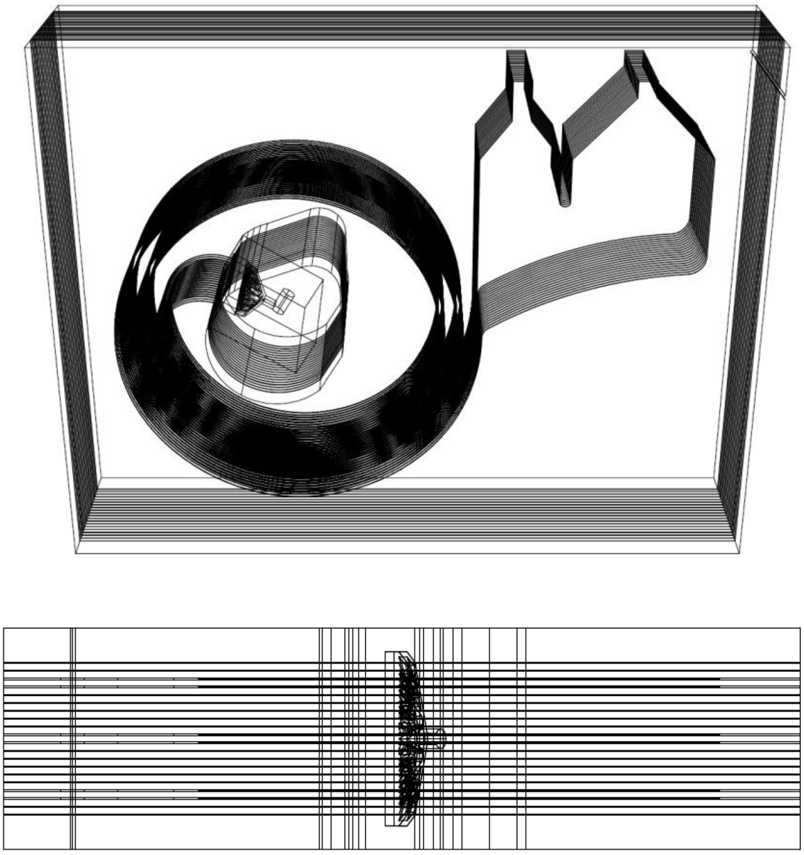

Figure S7. Stacked of 20 toroidal channels, 536 µm in height manufactured using a laser-cutter (*cf.* Table S3 and Fig. S1 for further details of a single toroidal channel).

	Red particles (38-45µm)	Blue particles (250-300µm)
Inlet	1.09 g	1.33 g

Table S6. Inlet concentrations for stack experiments with a starting volume of 7.05L

Video

In the video, red (Phosphorex, 250µm mean size) population of PS beads focused closed to the inner wall and collected in the focused outlet can be visualized with the naked eye (video recorded using a smartphone). The spiral channel is 500 µm high (1:6 aspect ratio with a rectangular cross-section) and the flowrate is 20 mL/min.

Supplementary Information References

1 Mach, A. J. & Di Carlo, D. Continuous scalable blood filtration device using inertial microfluidics. *Biotechnology and Bioengineering* **107**, 302-311 (2010).

2 Khoo, B. L. *et al.* Clinical Validation of an Ultra High-Throughput Spiral Microfluidics for the Detection and Enrichment of Viable Circulating Tumor Cells. *PLoS ONE* **9**, e99409 (2014).

3 Lee, W. *et al.* 3D-Printed Microfluidic Device for the Detection of Pathogenic Bacteria Using Size-based Separation in Helical Channel with Trapezoid Cross-Section. *Scientific Reports* **5**, 7717 (2015).

4 Warkiani, M. E. *et al.* Ultra-fast, label-free isolation of circulating tumor cells from blood using spiral microfluidics. *Nat. Protocols* **11**, 134-148 (2016).

5 Hur, S. C., Tse, H. T. K. & Di Carlo, D. Sheathless inertial cell ordering for extreme throughput flow cytometry. *Lab on a Chip* **10**, 274-280 (2010).

6 Zhang, J., Yan, S., Li, W., Alici, G. & Nguyen, N.-T. High throughput extraction of plasma using a secondary flow-aided inertial microfluidic device. *RSC Advances* **4**, 33149-33159 (2014).

7 Hansson, J. *et al.* Inertial microfluidics in parallel channels for high-throughput applications. *Lab on a Chip* **12**, 4644-4650 (2012).

8 Rafeie, M., Zhang, J., Asadnia, M., Li, W. & Warkiani, M. E. Multiplexing slanted spiral microchannels for ultra-fast blood plasma separation. *Lab on a Chip* **16**, 2791-2802 (2016).

9 Martel, J. M. *et al.* Continuous Flow Microfluidic Bioparticle Concentrator. *Scientific Reports* **5**, 11300 (2015).

10 Jimenez, M. & Bridle, H. Microfluidics for effective concentration and sorting of waterborne protozoan pathogens. *Journal of Microbiological Methods* **126**, 8-11 (2016).

11 Warkiani, M. E., Tay, A. K. P., Guan, G. & Han, J. Membrane-less microfiltration using inertial microfluidics. *Scientific Reports* **5**, 11018 (2015).

12 Guan, G. *et al.* Spiral microchannel with rectangular and trapezoidal cross-sections for size based particle separation. *Scientific Reports* **3**, 1475 (2013).

13 Kaltsas, G. *et al.* Focusing and Sorting of Particles in Spiral Microfluidic Channels. *Procedia Engineering* **25**, 1197-1200 (2011).
